# ﻿*Veronicahongii* (Plantaginaceae), a new species from Central China

**DOI:** 10.3897/phytokeys.220.96550

**Published:** 2023-02-24

**Authors:** Song-Zhi Xu, Qi-Liang Gan, Zun-Wei Ke, Zhen-Yu Li

**Affiliations:** 1 School of Life Science, Nantong University, Nantong 226019, Jiangsu, China Nantong University Nantong China; 2 Zhuxi Qiliang Biological Institute, Zhuxi 442300, Hubei, China Zhuxi Qiliang Biological Institute Hubei China; 3 Faculty of Biochemistry and Environmental Engineering, Hanjiang Normal University, Shiyan 442000, Hubei, China Hanjiang Normal University Hubei China; 4 State Key Laboratory of Systematic and Evolutionary Botany, Institute of Botany, Chinese Academy of Sciences, Beijing 100093, China Institute of Botany, Chinese Academy of Sciences Beijing China

**Keywords:** Central China, new species, taxonomy, *Veronicahongii*

## Abstract

A new species *Veronicahongii*, from western Hubei Province, Central China is described and illustrated. The species is morphologically similar to *V.henryi* Yamazaki, but mainly differs in the glabrous plant, except pedicels, broadly ovate leaf blades, glandular-pubescent pedicels, obovate calyx lobes, smaller corolla, broadly ovate capsule and much smaller seeds.

## ﻿Introduction

*Veronica* L. is a cosmopolitan genus consisting of ca. 250 species, mainly in Asia and Europe, of which 53 species are distributed in China (Hong and Fischer 1998). For a long time, *Veronica* has belonged to Scrophulariaceae (Fischer 2004). In recent years, *Veronica* L. has been transferred to Plantaginaceae (Albach et al. 2005; Angiosperm Phylogeny Group IV 2016). Some *Veronica* species have economic uses, including medicinal and ornamental value, while some other species are noxious weeds (Mabberley 1990). There are extremely rich *Veronica* species in Central China, including some endemic species, *Veronicaszechuanica* Batal., *V.fargesii* Franch., *V.henryi* Yamazaki and *V.laxissima* D.Y. Hong (Tsoong and Hong 1979; Hong 1996). Recently, an unknown *Veronica* species with some special characters was found during the fieldwork in Central China. The length of the seed of this species is only ca. 0.3 mm and should be the tiniest seed amongst species in *Veronica* (Thieret 1955; Martinez-Ortega and Rico 2001). The species is a terrestrial plant, with slender stems, axillary racemes, 4-parted calyx, rotated corolla, compressed and broadly ovate capsule and flattened seeds with convex both sides. After carefully checking related literature and specimens, we concluded this species should be placed in VeronicaSect.Veronica (Tsoong and Hong 1979) and it represents a species new to science. We describe and illustrate it here.

## ﻿Materials and methods

Specimens of the putative new species were collected in Zhuxi County of Hubei Province in 2022. Comparisons with its relatives were made by consulting specimens stored in PE or some virtual specimen databases (HIB, KUN, IBK, IBSC, CVH, JSTOR, CDBI and WUK). Morphological observations and measurements were based on living plants of four individuals in the field. All morphological characters were measured with dissecting microscopes and were described using the terminology presented by Harris and Harris (1994).

## ﻿Taxonomic treatment

### ﻿ Veronica hongii

Taxon classificationPlantaeLamialesPlantaginaceae

Q.L.Gan, Z.Y.Li & S.Z.Xu sp. nov.

F89A7E3F-7DF2-5190-A077-E8AF81172FFB

urn:lsid:ipni.org:names:77314713-1

Figs 1 , 2

#### Diagnosis.

*Veronicahongii* Q.L.Gan, Z.Y.Li & S.Z.Xu is similar to *V.henryi* Yamazaki in the perennial and diffuse plants, glabrous bracts and calyx and few-flowered racemes, but the new species can be easily distinguished from the latter by the glabrous plant, except pedicels, smaller leaf blades, corolla and seeds, longer and glandular-pubescent pedicels, obovate calyx lobes, broadly ovate capsule and flowering from September to October (see Table 1).

**Table 1.** Morphological comparisons of *Veronicahenryi* and *V.hongii*.

Characters	*V.henryi*	*V.hongii*
Stems	pubescent, becoming almost glabrous when old	glabrous
Leaf blades	ovate to narrowly ovate, 2–5 × 1.2–3 cm	broadly ovate, 0.6–1.8 × 0.4–1.3 cm
Pedicels	1–2 mm long at anthesis, 2–3 mm long in fruit, pubescent	3–5 mm long at anthesis, 5–7 mm long in fruit, glandular-pubescent
Calyx lobes	linear-lanceolate	obovate
Corolla	ca. 10 mm in diam., throat hairy	3.5–4 mm in diam., glabrous
Capsules	pliciform-rhomboid, 4–5 mm long, 9–11 mm wide, glandular-ciliate	broadly ovate, ca. 3 mm long and wide, glabrous
Seeds	ca. 1.5 mm long	ca. 0.3 mm long
Flowering	April to May	September to October

#### Type.

China. Hubei Province: Zhuxi County, Huiwan Town, Chuanfeng Village, on river bank, alt. 361 m, 22 September 2022, Q.L.Gan3312 (holotype, PE!; isotype, PE!).

#### Description.

Herbs perennial, plants diffuse. Stems terete, 5–18 cm long, 1.5–2 mm in diam., green or reddish-brown, glabrous, branched below the middle, branches slender, lower part prostrate and rooting at nodes, distally ascending, internodes 1.5–3 cm long. Leaves opposite, glabrous; petioles 2–6 mm long, lower ones longer, flattened, abaxial side shallowly grooved; leaf blades broadly ovate, 6–18 mm long, 4–13 mm wide, lower ones smaller, base broadly cuneate to rounded; margins shallowly serrate, crenate or subentire, apex acute to rounded; pinnately veined, mid-vein slightly impressed abaxially and prominent adaxially, lateral veins 2–3 on each side of mid-rib and alternate, veinlets inconspicuous. Racemes axillary from upper leaves, with 2–14 alternate flowers; peduncle 2–5 cm long, glabrous; axis 1–6 cm long, glabrous; bracts ovate-lanceolate to narrowly linear; pedicels filiform, straight or slightly incurved, 3–5 mm long at anthesis that elongate to 5–7 mm in fruit, sparsely with multicellular glandular hairs. Calyx glabrous 2–2.5 mm long, 4-parted, ca. 0.2 mm connated at base; lobes obovate, subequal, 1–1.3 mm wide. Corolla white and flushed purplish, with purple stripes, glabrous, rotated, 3.5–4 mm in diam., 4-parted; tube ca. 0.2 mm long; lower lobe smaller, obovate-rhombic, other 3 lobes rhombic, 2.5–3 mm long and wide, all lobes subacute at apex. Stamens 2, adnate to posterior side of corolla tube, slightly shorter than the lobes of corolla, glabrous; filaments white, 2–2.5 mm long; anthers purplish, ovate-oblong, ca. 0.9 mm long. Pistil glabrous; style ca. 2 times as long as the ovary; stigma capitellate; ovary rounded, slightly emarginate at apex. Capsule strongly compressed, broadly ovate, ca. 3 mm long and wide, glabrous, apex obtuse and small-notched, lateral angles rounded. Seeds 5–8, elliptic, ca. 0.3 mm long, flattened and convex on both sides, brown, glabrous.

**Figure 1.**
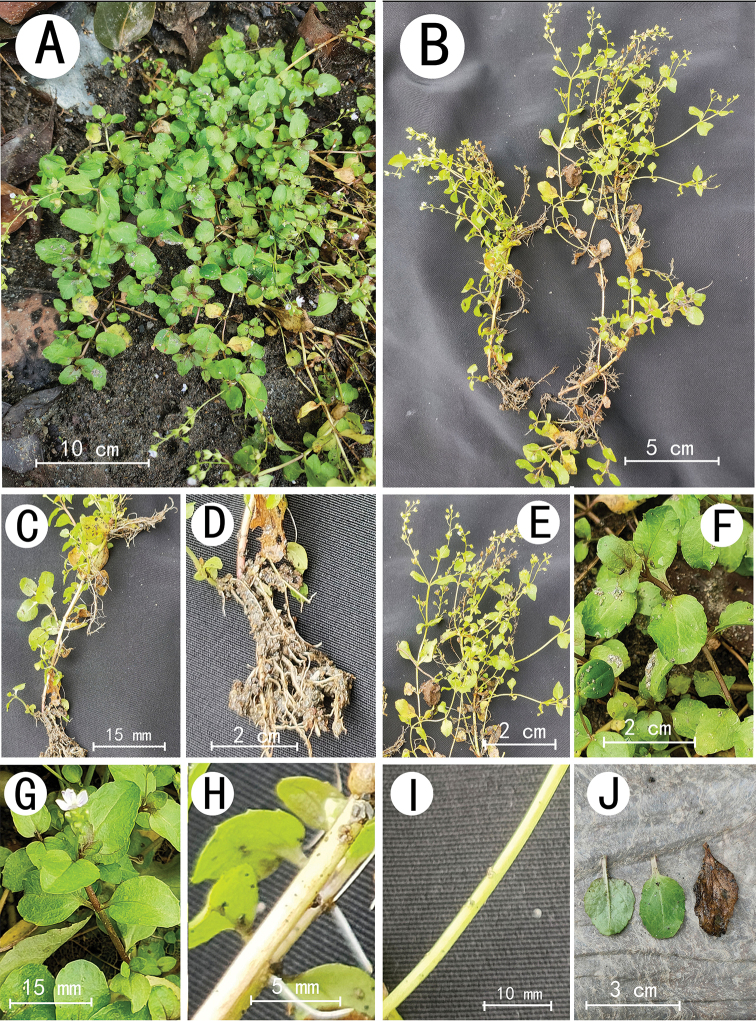
*Veronicahongii* sp. nov. **A, B** flowering plants **C, H** lower part of stem **D** roots **E, G** flowering branches **F** young branches **I** upper part of stem **J** leaf blades.

#### Phenology.

Flowering and fruiting from September to October.

#### Distribution and habitat.

The populations of *Veronicahongii* were known from Chuanfeng Village, Huiwan Town, Zhuxi County, Hubei Province. It grows in grassland on river banks at elevations ca. 361 m.

#### Etymology.

The species is named in honour of De-Yuan Hong (1937–), a famous botanist at the Institute of Botany, the Chinese Academy of Sciences (CAS), academician of CAS, who has devoted over 60 years to taxonomic and biosystematic studies of Paeoniaceae, Scrophulariaceae, Plantaginaceae, Campanulaceae, Commelinaceae and many other families, published *Plants of China* and *Flora of Pan-Himalaya*.

**Figure 2.**
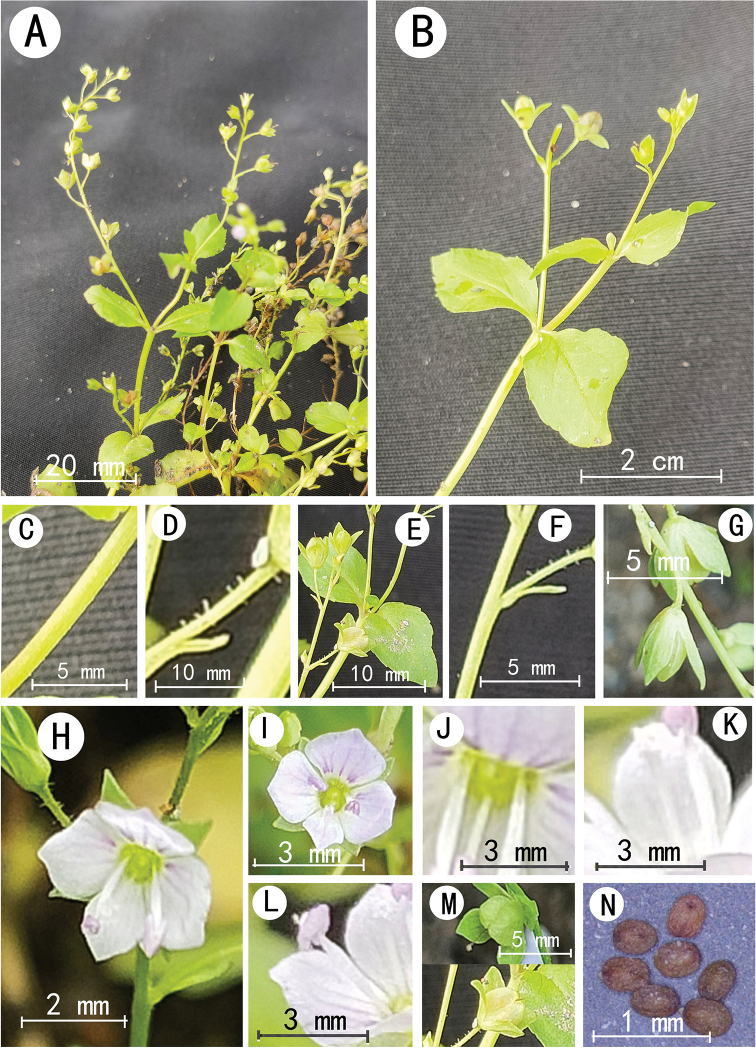
*Veronicahongii* sp. nov. **A–D** Fruiting branches **E** bract and pedicel **F** calyx **G** flower buds **H** flower **I** capsule **J** seeds.

#### Conservation assessment.

Based on the present field investigations, *Veronicahongii* is known from only one population composed of 11 individuals in Chuanfeng Village, Huiwan Town, Zhuxi County, Hubei Province. The provisional conservation status is Critically Endangered (CR), based on criteria D (number of mature individuals fewer than 50) (IUCN 2022).

#### Paratypes.

China. Hubei Province: Zhuxi County, Huiwan Town, Chuanfeng Village, on river bank, alt. 361 m, 22 September 2022, Q.L.Gan3313 (PE!).

## Supplementary Material

XML Treatment for Veronica hongii
